# Abilities and Disabilities Among Children With Developmental Language Disorder

**DOI:** 10.1044/2023_LSHSS-22-00070

**Published:** 2023-05-02

**Authors:** Karla K. McGregor, Nancy Ohlmann, Nichole Eden, Tim Arbisi-Kelm, Alys Young

**Affiliations:** a Boys Town National Research Hospital, Omaha, NE; b The University of Manchester, United Kingdom

## Abstract

**Purpose:**

The aim of this study was to situate developmental language disorder (DLD) within the impairment and disability framework of the International Classification of Functioning, Disability, and Health (ICF); describe the functional strengths and weaknesses of a cohort of first-grade children with DLD and their peers; and explore the ways that language-related disabilities relate to language impairment, developmental risk, and receipt of language services.

**Method:**

We queried the caregivers of 35 children with DLD and 44 peers with typical language development about their children's language-related functions, developmental risks, and language services using mixed quantitative and qualitative methods.

**Results:**

The children with DLD presented with weaknesses in domains that are highly dependent upon language skill, including communication, community function, interpersonal relationships, and academics. They presented with strengths in domestic and personal aspects of daily living, play and coping aspects of socialization, and gross motor function. Caregivers of children with DLD expressed pride in their children's agentive and prosocial qualities. Consistent with the ICF, what distinguished children with DLD who had functional weaknesses and disabilities from those who had healthy function was not the severity of language impairment as measured by decontextualized tests of language skill, but the presence of cumulative developmental risks. Compared to those with healthy function, a larger portion of children with weaknesses and disabilities were receiving language services; however, two girls who had disabilities despite mild levels of impairment were without services.

**Conclusions:**

Children with DLD present with predictable strengths and weaknesses in everyday language-related functioning. For some children, the weaknesses are mild, but for others, they limit function to a greater extent and should be considered disabilities. The severity of language impairment is not a strong indicator of language-related function and, therefore, is not a good metric for determining service qualification.

Developmental language disorder (DLD) is a neurodevelopmental condition that impairs the learning, expression, and comprehension of spoken and written language in people of all ages (Bishop et al., 2017). The problem is not the result of intellectual disability, hearing impairment, autism, or any other diagnosable condition known to limit language function. Instead, it arises over the course of early development from a complex and, as yet, poorly understood mix of polygenic and environmental risks (Mountford et al., 2022; Onnis et al., 2018). Prevalence estimates hover around 7% (Norbury et al., 2016; Tomblin et al., 1997).^1^

Language is essential to human life. We use language to engage with friends, order food in a restaurant, complete an exam, or apply for a job. Thus, it is unsurprising that people with DLD tend to function more poorly than matched peers in everyday social, community, academic, and professional settings. In some but not all individuals, these limitations on function constitute a disability. In this article, we distinguish impairment from disability and explore factors that lead some children with DLD to experience disability, while others do not.

The extent of the disability matters when qualifying for and receiving language treatment. For example, under the provisions of the Individuals with Disabilities Education Improvement Act of 2004 (IDEA, 2004), children with DLD qualify for special education services in U.S. schools at no cost only when they (a) have a language impairment that (b) interferes with educational function and that (c) requires special instruction (34 CFR § 300.8). Thus, given criterion 2, only children experiencing disability in the school setting will qualify for services; impairment alone is not enough. Nevertheless, IDEA is interpreted and operationalized differently across the states. Therefore, a better understanding of the disabilities associated with DLD and the relationship between language impairment and disability may help to ensure that individuals who need language services and related supports are not overlooked.

## Impairment and Disability Defined

We frame this study within the guidelines of the International Classification of Functioning, Disability, and Health (ICF; World Health Organization, 2001). The ICF defines *impairment* as a significant limitation in the structure or function of the body. In the case of DLD, we can be more specific: DLD reflects impairment in brain function and associated psychological processes that are not the result of injury or illness but that emerge with development (Krishnan et al., 2016; Lee et al., 2020). Impaired language skills and weak language skills exist on a continuum. For example, a child could be relatively weak in the use of complex sentence structures compared to other children, but that weakness is not significant enough to be considered an impairment. Standardized tests help speech-language pathologists (SLPs) determine whether low language performance is a weakness or an impairment and, in the latter case, quantify severity. These tests reveal what the child can do on decontextualized tasks that probe language skill.

The ICF defines *disability* as a significant limitation on activities and participation in everyday life. Disabled language-related functions and weak language-related functions also exist on a continuum. For example, a child could have more difficulty with language-heavy academic tasks than other children, but that weakness may not be significant enough to be considered a disability. To identify disabilities, SLPs use functional measures such as language samples, classroom observations, and caregiver or teacher surveys to probe what the child does do with language to accomplish social, academic, domestic, and community functions on a daily basis.

The ICF acknowledges the role of the biological impairment and associated psychological processes in limiting function while affirming the critical role of society—its values, biases, policies, and institutions—in the ultimate determination of functional outcomes. In other words, disability is the dynamic product of internal and external factors: the biology and psychology of the child and the social, cultural, and structural world around the child (Washington, 2007). Each individual will experience a unique and complex mix of internal and external factors, and the mixture can increase the risk of disability or protect from disability.

Given this biopsychosocial model of disability, one would predict that language impairment alone will not cause language-related disabilities, but language impairment in the context of high cumulative risks (or few protective factors) will. In other words, if two children, A and B, have language impairments that are identical in severity but A experiences many more risks to healthy function, A is more likely to have a language-related disability than B. Moreover, the ratio of risks to protective factors is likely to vary over developmental time; in other words, the different functional levels represented by A and B could be realized in the same child at different ages.

## Risks and Protective Factors Associated With DLD

### Risks

One cannot anticipate the myriad of risks that any given individual might face. However, we can draw from what we already know about DLD at the population level to predict some likely candidates. Here we highlight three: low nonverbal IQ (NVIQ), low caregiver educational attainment, and other health conditions.

#### NVIQ

By definition, children with DLD may have high, average, or below-average nonverbal cognitive abilities as long as the below-average abilities are not so low as to constitute an intellectual disability (Bishop et al., 2017). Nevertheless, skill levels in verbal and nonverbal domains are positively correlated; thus, children with DLD tend to have lower NVIQ than children with typical language skills (Gallinat & Spaulding, 2014). Although low NVIQ does not limit these children's rate of language growth (Norbury et al., 2017), it could constitute an internal barrier to function in the moment. In fact, NVIQ, but not measures of expressive language, correlated positively with communicative function in a sample of preschoolers with DLD (Hall & Segarra, 2007).

#### Caregiver Educational Attainment

Like all vulnerable children, those with DLD who live in low-socioeconomic circumstances are at heightened risk for disabled function because of broad effects on family stress and agency and a range of intersecting social determinants including quality of schools, safety of communities, access to nutritious food and health care, and opportunities for experiences that promote learning and development (Adler & Snibbe, 2003; Murray, 2003). Low socioeconomic status (SES) is also associated with long-standing institutional racism and social exclusion. Thus, for many children, these ill effects are compounded by discrimination (Terry et al., 2022).

Caregiver educational attainment and household income are frequent proxies for the measurement of SES, but among these correlated variables, caregiver education is the stronger predictor of children's cognitive and academic outcomes (Davis-Kean et al., 2021) and the stronger predictor of DLD (Rudolph, 2017). A child with DLD is about twice as likely to have a mother with less than a high school education than a child with typical language development (TD; Rudolph, 2017). Although caregivers do not cause DLD, the circumstances associated with low educational attainment such as reduced financial resources and reduced sociocultural capital may constitute an external barrier to a vulnerable child's language-related function.

#### Health

Poor health may increase the risk of disability. Health conditions result not only in direct effects like pain, discomfort, or fatigue but also in indirect effects like stress and financial challenges. Certain health conditions are known to be more prevalent among individuals with DLD than in the general population. For example, compared to unaffected peers, individuals with DLD are 2–3 times more likely to have attention-deficit/hyperactivity disorder (ADHD; Mueller & Tomblin, 2012), 6 times more likely to have clinical anxiety, and 3 times more likely to have clinical depression (Conti-Ramsden & Botting, 2008). Approximately one-third of children who have DLD also meet diagnostic criteria for developmental coordination disorder (Flapper & Schoemaker, 2013). Oral motor skills may also be weak: Medical record reviews suggest that nearly 50% of children with DLD have early feeding and swallowing difficulties (Malas et al., 2017).

In summary, the extant literature suggests that children with DLD are more likely to have lower NVIQs, have caregivers with low educational attainment, and experience poor health than children who are neurotypical. Here, we hypothesize that the presence of these risks may increase the likelihood of language-related disability among those with language impairment by worsening its severity or by introducing environmental barriers at the biological, psychological, familial, and societal levels. We cannot fully disentangle risks that make the impairment itself more likely from those that make disability more likely. Moreover, there are other risk factors associated with DLD, such as exposure to tobacco smoke during gestation (Calder et al., 2022; Tomblin et al., 1998), that we could examine. We chose to focus on low NVIQ, low caregiver educational attainment, and the presence of other health conditions because of their potentially broad and long-lasting impacts on the contexts in which children function.

### Protective Factors

Risks can be offset by protective factors, and the child's individual strengths can serve as internal protection (World Health Organization, 2001). Consider, for example, that many children with DLD are highly prosocial, meaning that they tend to behave in a kind, considerate, or helpful manner (Farmer, 2000, but see Fujiki et al., 1999). Prosociality is a trait known to buffer individuals with DLD from social (Mok et al., 2014), emotional (Conti-Ramsden et al., 2018), and behavioral (Pickles et al., 2016) dysfunction.

When interviewed by Lyons and Roulstone (2018), 9- to 12-year-olds with DLD often shared how they had overcome problems to achieve a positive outcome. These recounts suggest three additional interrelated factors that may be protective: agency, coping, and resilience. Agency refers to the child's active role in solving problems, whereas coping refers to their ability to regulate thoughts, emotions, and behaviors when faced with a problem (Compas et al., 2001). These inner resources help the child to build resilience, that is, the capacity to adapt successfully to stressful situations (Chatterjee, 2018;). In other words, resilience emerges in response to risk (Rutter, 2006) and, in turn, may serve to counter the impact of future risks.

Resilience, like disability, does not reside solely within the child. Positive role models, reliable structure, and appropriate support within the family or broader community are critical ingredients in the child's ability to demonstrate resilience (ten Hove & Rosenbaum, 2018). Thus, it is notable that the children interviewed by Lyons and Roulstone (2018) also emphasized the importance of warm relationships with family, friends, and pets as crucial to their well-being.

## Disabilities Associated With DLD

Individuals with DLD may be disabled in various aspects of everyday language-related functions, including communication (e.g., Lyons & Roulstone, 2018; Rost & McGregor, 2012), socialization (e.g., Brinton et al., 1997, 1998; Mok et al., 2014; Rice et al., 1991; Timler, 2008), and academics (e.g., Dockrell et al., 2011; Norbury et al., 2016; Young et al., 2002). Albeit for valid reasons, studies of language-related function among people with DLD tend to focus on one aspect or another, making it difficult to know the extent to which multiple disabilities are present within a given individual or even within a given cohort.

Two notable exceptions to this piecemeal approach are studies by Croteau et al. (2015) and Shevell et al. (2005). Croteau et al. (2015) administered the Assessment of Life Habits Questionnaire (Fougeyrollas et al., 2001) to the parents and teachers of children with DLD who were 5–13 years old. All children were in receipt of language services from an SLP who judged their language impairments to be severe. Parents and teachers agreed that the children's most notable strengths were personal care, mobility, and play. Their most limited functions occurred in the domains of interpersonal relationships, communication, academics, and responsibility (e.g., taking care with money and following safety practices). Whether these weaknesses were severe enough to be considered disabilities was not evaluated.

Shevell et al. (2005) administered two caregiver-report measures of communication, the Battelle Developmental Inventory (BDI; Newborg et al., 1984) and the Vineland Adaptive Behavior Scales (VABS; Sparrow et al., 1984), to identify impairment (via the BDI) and disability (via the VABS) among 42 seven-year-old children who had been previously diagnosed with DLD as preschoolers by a clinical team at Montreal Children's Hospital. Given a cutoff score of −1.5 *SD*s below the mean on the BDI, 83% continued to demonstrate impairment at the age of 7 years. However, as expected, given the ICF biopsychosocial model, fewer—only 64%—had a disability in one or more language-related domains of function: 48% in communication, 45% in daily living, and 19% in socialization.

A final outcome is noteworthy. Although 64% of the children demonstrated a disability in at least one domain, only 35% received services at school. Thus, in this sample, many children with disabilities were not receiving interventions or other supports that could improve their participation in everyday life. This troubling observation punctuates earlier findings by Tomblin et al. (1997) and later findings by Norbury et al. (2016) documenting a low rate of services among children with DLD. Girls, children from marginalized groups, children from low-SES families, and children who present without other, more obvious conditions (e.g., speech-sound disorders) are especially likely to go without services (Bishop & McDonald, 2009; Calder et al., 2022; Morgan et al., 2017; Wittke & Spaulding, 2018). These reports serve as additional motivation to explore the disabilities associated with DLD.

## This Study

This study focused on the function of first-grade children with DLD or TD. The first year of formal schooling marks a transition to longer, more structured days; a more formal academic focus; and, likely, a larger peer group. With these increased contextual demands, we reasoned that first grade is a crucial point to explore disability among children with DLD. Moreover, most first-graders in the United States are 6 or 7 years old; thus, we were able to compare our results to the results reported by Shevell et al. (2005).

Like Shevell et al. (2005), we used the VABS (in our case, the 3rd edition; Sparrow et al., 2016) to establish a profile of language-related function. However, we extended their work in six ways. First, in recognition of the contextualized nature of disability, we included a comparison group of children with TD drawn from the same communities as the children with DLD. Doing so allowed us to find patterns of relative strengths and weaknesses in function across a broad spectrum of language skill. Second, we included the optional motor domain questions, to determine the status of motor function in the DLD profile. Third, we probed communication and academic function by including relevant questions in a clinical history intake completed by the caregivers. Fourth, in that same intake, we queried demographics and health history to determine whether low caregiver educational attainment and the presence of other health conditions accompanied disability. Fifth, we measured NVIQ to determine whether low scores contributed to the risk of disability. Sixth, we appended a narrative prompt to the VABS-3 interview. We asked caregivers to tell us about a moment when they were proud of their child. In analyzing their responses, we hoped to gain insights into particular strengths (potential protective factors) among children with DLD through the perspective of their caregivers.

We analyzed the data using mixed methods: quantitative comparisons of the VABS-3 scores and clinical intake questions and qualitative analysis of the proud caregiver narratives. Mixed methods are optimal for exploring complex psychosocial phenomena like disability (Malina et al., 2011; Odom et al., 2006). With the rich description and case comparisons enabled by the qualitative approach, we aimed to ground the general understanding of disability derived from the quantitative approach in the experiences of individuals who live with DLD. We used the outcomes to address five main questions.

1. What strengths characterize this cohort of children with DLD? We predicted strengths in the psychosocial domain, especially in the areas of prosocial behavior, agency, and coping.
2. What weaknesses characterize this cohort of children with DLD? We predicted weaknesses in communication, daily living, social relationships, and academics. In addition, we predicted that for many children, weaknesses would be extreme enough to constitute a disability. We predicted that disability would be particularly prevalent in the domains of communication and academics.
3. What is the relationship between disability and impairment? Given the ICF biopsychosocial model and the results of Shevell et al. (2005), we predicted that some but not all children with language impairment would present with language-related disabilities and that those with more severe impairment would be more likely to present with disability than those with less severe impairment.
4. What is the relationship between disability and developmental risk? We predicted that children with language-related disabilities would be more likely than other children to present with three risk factors: low NVIQ, low caregiver education, or other health conditions.
5. What is the relationship between disability and receipt of services? Given the qualification criteria in IDEA, we predicted that children with language impairment plus language-related disability would be more likely to receive services than children with impairment alone.

## Method

### Ethics

This study was approved by the institutional review board of Boys Town National Research Hospital, approval number 17–04-XP. The participants gave informed assent/consent before taking part. The data were collected between January 30, 2018, and September 23, 2020.

### Participants

#### Caregivers

The caregivers of 79 first graders (ages 6.2–8.1 years), 35 with DLD and 44 with TD, were interviewed. The sample size was a matter of convenience: These caregivers were available to us because their children were enrolled in a longitudinal study of word learning (the Dynamics of Word Learning; Karla K. McGregor, Principal Investigator).

In the DLD group, the participating caregivers were 29 mothers, four fathers, one grandmother, and one aunt. The grandmother and aunt had legal custody, and the children lived with them permanently. Twenty-nine were White, four were Black, and two did not report. One person was Hispanic. They averaged 14.10 years of education (*SD* = 2.26). In the TD group were 39 mothers and seven fathers; in two cases, the mother and father participated. Thus, there were a total of 46 caregivers for 44 children. Forty-three were White, one was Black, one was Asian, and one did not report. No one was Hispanic/Latinx. They averaged 16.63 years of education (*SD* = 2.33). Years of education differed between groups favoring the TD group, *t*(1, 79) = −4.88, *p* < .0001, Hedges's *g* = 1.10.

#### Children

The children were recruited via language screenings conducted in schools (DLD: *n* = 19; TD: *n* = 38) or the community (DLD: *n* = 3; TD: *n* = 2), media advertisements (DLD: *n* = 7; TD: *n* = 1), or word of mouth (DLD: *n* = 5; TD: *n* = 3). Most lived in Iowa (DLD: *n* = 26; TD: *n* = 40) or Nebraska (DLD: *n* = 7; TD: *n* = 3), but those who enrolled after the onset of the COVID-19 pandemic hailed from other U.S. locations as well (North Carolina and Arkansas; DLD: *n* = 2; TD: *n* = 1), enabled by the use of zoom for remote data collection.

All participants in the DLD group scored below the 15th percentile on the Redmond Sentence Recall task (Redmond et al., 2019) and below a standard score of 92 on the Test of Narrative Language–Second Edition (TNL-2, Gillam & Pearson, 2017). Those with TD scored above these cutpoints. Ninety-two is the cutpoint that maximizes the sensitivity and specificity of DLD diagnosis on the TNL-2 (Gillam & Pearson, 2017). The composite scores on the TNL-2 ranged from 61 to 91 in the DLD group and 94 to 127 in the TD group. In other words, although we categorized children's language skills as impaired or typical as has been traditional in the research literature, their performance on this language measure represented a continuous distribution in accordance with the ICF model and with current conceptualizations of DLD (Lancaster & Camarata, 2019).

To participate in the longitudinal study, all children had to be English learners from birth; pass an audiometric pure-tone screening by responding at 25 dB or lower at 0.5, 1, 2, and 4 kHz, bilaterally; and score higher than a standard score of 69 on the perceptual composite of the Wechsler Abbreviated Scale of Intelligence–Second Edition (WASI-II; Wechsler, 2011). The WASI-II perceptual composite also served as our proxy for risk associated with low NVIQ, which we defined as a standard score of 70–84, inclusive. All children had to be in first grade (finished kindergarten and not begun grade 2). No child had a diagnosis of autism spectrum disorder, genetic syndromes, or neurological insult via caregiver report. Given that ADHD is often comorbid with DLD (Sciberras et al., 2014), we did not exclude children who had ADHD diagnoses. These were six children in the DLD group (17%) and three in the TD group (7%), χ^2^(2, *N* = 79) = 2.06, *p* = .15.

One additional child had been recruited for the DLD group but was diagnosed with epilepsy after the caregiver interview. Therefore, the data for that child were excluded here, reducing the total number of children with DLD to 35.

### Materials

#### VABS-3

VABS-3 is a measure of adaptive behavior published in 2005. It was standardized on a geographically diverse sample of 2,560 people that reflect the educational, racial, and ethnic demographics of the United States. We administered the comprehensive interview version covering three domains of language-related function: Communication: receptive, expressive, and written; Socialization: interpersonal relationships, play and leisure, and coping skills; and Daily Living: personal, domestic, and community. We also included the optional Motor domain: gross and fine. Most of the children in our sample were 7 years old. At that age, the internal consistency of the interview form ranges from .99 (adaptive behavior composite) to .76 (gross motor subdomain). Test–retest correlations range from .89 (communication) to .73 (coping skills) and inter-examiner correlations range from .77 (community) to .61 (play). The concurrent validity is demonstrated by correlations between the VABS-3 Parent Interview Form and independent measures of adaptive behavior that range from *r* = .41 to .98 (Pepperdine & McCrimmon, 2018). As for content validity, raw scores increase with age in a manner consistent with expected developmental trends.

When administering this test, the examiner asks the caregiver prespecified open-ended questions to prompt a description of the child's behavior in the developmental domain under consideration. Given that description, the examiner assigns a score of 0 (*never*), 1 (*sometimes*), or 2 (*usually or often*) for that item and tallies a total score per subdomain. There were 458 items in the domains we tested; however, the interview questions are arranged developmentally with basals and ceilings, so the total number of items administered varied across caregivers.

#### Case History Intake

The case history intake provided data on risk factors. We determined the presence of other health conditions by parental endorsement of any of the following diagnoses: ADHD, anxiety, arthritis, balance problem, behavior disorder, depression, feeding and swallowing problem, fine motor problem, gross motor problem, heart condition, kidney condition, muscle weakness, sensory processing disorder, skeletal anomaly, sleep disorder, thyroid condition, and vision problem (that could not be corrected by glasses).

Case history questions about current communication and academic function supplemented the VABS-3 outcomes. Specifically, caretakers noted the extent to which their children avoided talking, cried, or became aggressive in response to communication breakdowns. Caretakers rated their children's academic performance as below average, average, or above average. They indicated any concerns about their children's ability to complete school assignments, and they shared impressions of the children's learning abilities.

Caregivers reported their years of education. We defined low attainment as less than 13 years (Condliffe & Link, 2008). This binary classification (low/not low) is admittedly blunt, but we lacked a large enough sample to perform a more nuanced evaluation. With a small sample, greater degrees of classification could of itself produce errors in interpretation. Finally, we asked whether the child received language services (for spoken or written language but not articulation only) and whether the provider of those services was an SLP.

### Procedure

We interviewed the caregivers in their homes, a laboratory van parked outside their homes, or the research laboratory, according to their preference. The final five enrolled caregivers participated remotely because the COVID-19 pandemic prevented a face-to-face interview.

The examiner was one of three experienced researchers: two female SLPs and one male linguist. In each case, the examiner had established familiarity and rapport with the caregiver in three prior visits when the child had participated in the Dynamics of Word Learning sessions. The examiners followed the administration instructions in the VABS-3 manual with fidelity exceeding 95%, as determined by an independent researcher who listened to 20% of the interviews. All interviews were conducted in spoken English.

The session was recorded using an external Snowball Blue USB microphone and saved in an mp3 format. The session began with a formal interview structured by the administration of VABS-3. Afterward, the examiner asked the caregiver to “Tell me about a time when you were especially proud of your child.” If the caregiver did not expound, the examiner asked, “What specifically made you proud?” The session lasted 1–2 hr.

### Data Scoring, Coding, and Analysis

The examiner scored VABS-3 as directed in the manual. When 20% of the interviews were rescored by an independent researcher, agreement averaged 97%. We analyzed the VABS-3 outcomes in three ways. First, we took advantage of the VABS-3 automatic scoring program to conduct a within-individual, criterion-referenced comparison of relative strengths and weaknesses. Specifically, the program compares a given child's scores on each subdomain to their mean score and identifies statistically significant (*p* = .10) divergences from that mean. It is critical to keep in mind that this is a criterion-referenced measure: The reference here is the child, not the population; therefore, a *v* score of 14, for example, could represent a relative strength for one child, and that same score could represent a relative weakness for another. Moreover, a strength for a given child might not constitute a strength relative to age expectations.

Second, we used the VABS-3 norms to determine the standard scores earned in each domain and subdomain by each participant. We compared the scores of the DLD and TD groups via *t* tests for independent samples.

In the third comparison, instead of examining mean performance, we focused on the percentage of children in each group who demonstrated functional problems in communication, daily living, and socialization, operationalized as scores that indicated subclinical weaknesses (between −1 and −1.5 *SD* from the mean) or disabilities (scores that fell more than −1.5 *SD* from the mean). We specified these cutpoints to enable comparison to Shevell et al. (2005).

VABS-3 does not probe academic function directly. To address this gap, we operationalized academic disability as a parental endorsement of both “below average academic performance” and “difficulty with school assignments” on the case history OR a score on the written language subdomain of VABS-3 of 9 or lower (i.e., poorer than 1.5 *SD* below the mean). We considered an endorsement of either “below average academic performance” or “difficulty with school assignments” OR a written language score of 10 or 11 as indicative of academic weakness.

We categorized each child by functional ability in the domains of communication, daily living, socialization, and academics as follows:

1. healthy in all domains (no disabilities or weaknesses);
2. weak in a single domain (no disabilities);
3. weak in more than one domain (no disabilities);
4. disabled in one domain (no weaknesses);
5. disabled in one domain plus one or more weaknesses;
6. disabled in two domains (no weaknesses); and
7. disabled in two domains plus additional weaknesses or disabilities.

We transcribed the proud caregiver narratives verbatim into word.docx documents and replaced any names with the letters A, B, C, and so on. We included fillers and pauses in the caregiver narratives and the infrequent comments of the examiner to ensure an adequate context for interpreting emotions and nuanced meanings. A research assistant independently transcribed 10 randomly selected narratives. To reduce bias, this assistant was blind to the participants' group membership. Word-to-word agreement between the first and second transcripts ranged from 90% to 98% and averaged 95%. Caregivers of children with DLD gave shorter answers (*M*_number of words_ = 223, *SD* = 128) than caregivers of children with TD (*M*_number of words_ = 360, *SD* = 219), *t* = −2.37, *df* = 77, *p* = .0016, Hedges's *ɡ* = .74. Although we worked from these transcripts, the recordings remained available and we at times went back to them to check our interpretation of the caregivers' narratives.

The first author, an experienced pediatric research SLP, and the second author, an experienced pediatric clinical SLP, conducted the qualitative analysis. Our experiences as SLPs meant that we brought expertise to the analysis but also likely biases that influenced our interpretations of the data. Our methodological orientation was one of interpretive phenomenology (Neubauer et al., 2019). That is, we sought a further understanding of the phenomena of developmental language abilities and disabilities by interpreting the narrative responses of adults whose lived experience includes parenting a child with DLD.

Analysis was both deductive and inductive. We first generated a priori themes based on previous literature that we anticipated to be related to caregiver pride: the child's agency; prosocial behavior; and accomplishments in academics, athletics, or the arts. Next, inductive themes emerging from a close reading of the transcripts were added by the second author. Codes at the superordinate and subordinate levels were entered into the NVivo 12-Plus software permitting flexible and generative coding when new themes emerged, or a priori themes became complex. The first author then reviewed the themes and collapsed some to simplify patterns in the data. For example, all achievements in art had to do with dance, so we collapsed arts and athletics. The final themes were as follows:

1. accomplishment, with subthemes of academics, athletics, achieving goals, and overcoming obstacles;
2. agency, with subthemes of independence, confidence, and courage;
3. prosociality, with subthemes of empathy, generosity, helpfulness, and protecting family;
4. obeying rules; and
5. religious faith.

Single pieces of text could be given multiple codes if more than one theme was present, and single codes could encompass large sections of narrative material. We linked the themes, where relevant, to the developmental domains on the VABS-3. For example, many of the moments that involved academic achievement had to do with the child's reading ability, which links to the written language subdomain. The purpose of coding themes according to the VABS-3 domains was to facilitate data triangulation.

Finally, we added codes for the setting of the proud moments, those being home, school, or other. Where relevant, group comparisons were accomplished via chi-square tests.

## Results

### Strengths

**Figure 1.**
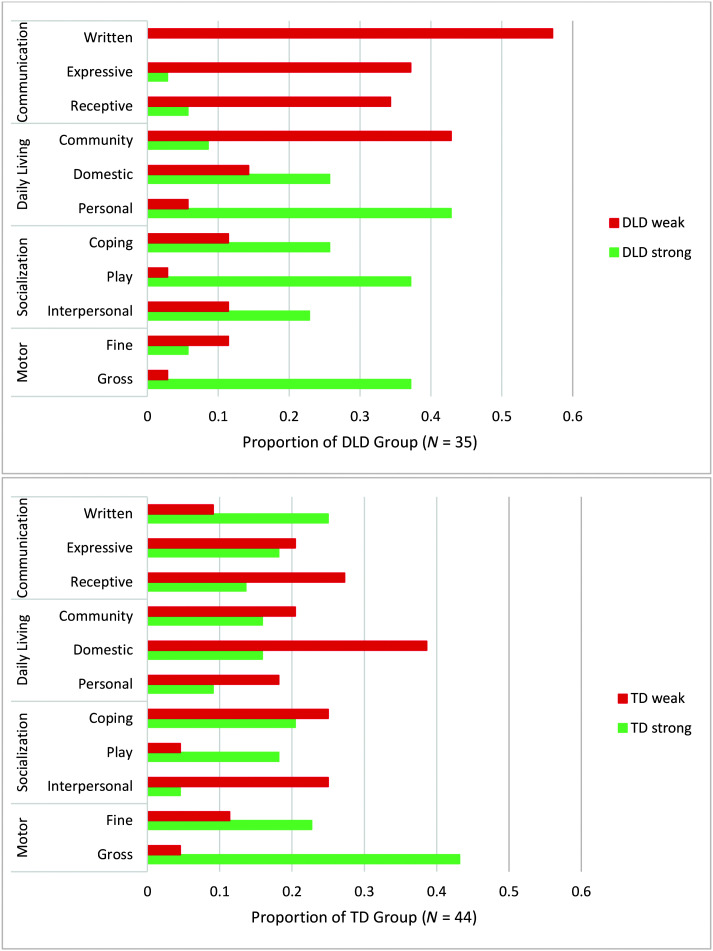
Relative strengths and weaknesses of the children with developmental language disorder (DLD, top) and typical language development (TD, bottom) as indexed by v scores on the Vineland Adaptive Behavior Scales-3.

Among the children with DLD, the domestic and personal aspects of daily living; the coping, play/leisure, and interpersonal aspects of socialization; and gross motor function more often constituted relative strengths than weaknesses (see Figure 1). Except for the interpersonal subdomain, the normative comparisons confirmed these as strengths at the group level (see Table 1): The DLD group performed near the expected mean and no differently from the TD group in these areas.

**Table 1.** Comparison of the profiles of DLD and TD groups on the Vineland Adaptive Behavior Scales-3.

Domain	Score comparison					Weaknesses				Disability			
	DLD (*n* = 35), *M* (*SD*)	TD (*n* = 44), *M* (*SD*)	*t* (*df* = 77)	*p*	Hedges' *g*	DLD		TD		DLD		TD	
						*n*	(%)	*n*	(%)	*n*	(%)	*n*	(%)
**Adaptive composite**	88.34 (9.03)	100.50 (9.24)	−5.87	< .00001*	−1.32	10	(29)	1	(2)	2	(6)	0	(0)
**Communication composite**	84.14 (9.54)	102.09 (8.47)	−8.85	< .00001*	−1.98	10	(29)	1	(2)	6	(17)	0	(0)
Written	11.71 (1.95)	15.61 (2.05)	−8.59	< .00001*	−1.92	10	(29)	0	(0)	4	(11)	0	(0)
Expressive	12.29 (2.62)	15.43 (1.77)	−6.35	< .00001*	−1.42	7	(20)	0	(0)	2	(6)	0	(0)
Receptive	13.03 (2.28)	15.11 (1.77)	−4.58	.000018*	−1.02	7	(20)	0	(0)	2	(6)	0	(0)
**Daily living composite**	93.06 (10.72)	99.68 (11.31)	−2.65	.009873	−0.59	3	(9)	4	(9)	2	(6)	0	(0)
Community	12.89 (2.47)	15.16 (2.19)	−4.33	.000044*	−0.97	5	(14)	3	(7)	4	(11)	0	(0)
Domestic	14.11 (2.49)	14.75 (2.79)	−1.05	.295069	−0.24	6	(17)	4	(9)	0	(0)	1	(2)
Personal	14.57 (2.17)	15.09 (1.84)	−1.15	.253643	−0.26	2	(6)	1	(2)	1	(3)	0	(0)
**Socialization composite**	95.51 (9.26)	100.98 (7.41)	−2.91	.004662	−0.65	5	(14)	2	(5)	0	(0)	0	(0)
Interpersonal	13.86 (1.52)	14.88 (1.33)	−3.21	.001962*	−0.71	2	(6)	1	(2)	0	(0)	0	(0)
Play	14.63 (1.90)	15.66 (1.58)	−2.63	.010244	−0.59	1	(3)	0	(0)	0	(0)	0	(0)
Coping	14.23 (1.99)	14.93 (1.78)	−1.66	.101961	−0.34	1	(3)	1	(2)	1	(3)	0	(0)
**Motor skills composite**	96.17 (11.28)	104.93 (10.03)	−3.65	.000479*	−0.82	1	(3)	1	(2)	2	6	0	(0)
Fine	13.60 (2.26)	15.16 (1.83)	−3.39	.001120	−0.76	4	(11)	0	(0)	1	3	0	(0)
Gross	15.14 (2.25)	16.39 (1.96)	−2.63	.010433	−0.59	2	(6)	1	(2)	0	0	0	(0)

*Note.* Subclinical weaknesses = scores that fall between −1 *SD* to −1.5 *SD*s below the mean. On the composites, this equates to scores of 84–77, inclusive (*M* = 100, *SD* = 15); on the subtest scores, this equates to scores from 11 to 10, inclusive (*M* = 15, *SD* = 3). Disability = scores that fall more than −1.5 *SD*s below the mean. On the composites, this equates to scores lower than 77; on the subtest scores, this equates to scores lower than 10. DLD = developmental language disorder; TD = typical language development.

* With a Bonferroni correction for multiple comparisons, alpha < .003.

As we will illustrate in the next sections, woven throughout the DLD caregiver narratives were examples of agentive, prosocial behaviors, or accomplishments that mapped onto the strengths found in their children's VABS-3 profiles (see Table 2). In fact, the DLD and TD caregiver groups did not differ significantly in frequency of proud moments that related to activities of daily living, χ^2^(2, *N* = 79) = 1.8, *p* = .17, socialization, χ^2^(2, *N* = 79) = 2.75, *p* = .10, or gross motor function, χ^2^(2, *N* = 79) = 0.33, *p* = .07. We take this as additional evidence that the children with DLD were by and large functioning age-appropriately in these areas.

**Table 2.** Caregiver narrative coding categories and results.

Codes	Description	DLD (*n* = 35)		TD (*n* = 44)	
		*n*	(%)	*n*	(%)
Developmental domains mentioned in caregiver narratives					
Communication		3	(9)	16	(36)
Written	Using reading or writing	0	(0)	14	(32)
Expressive	Producing spoken language	2	(6)	4	(9)
Receptive	Comprehending spoken information	1	(3)	0	(0)
Daily living		9	(26)	6	(14)
Community	Functioning outside the home: money, safety, and responsibilities	0	(0)	1	(2)
Domestic	Performing household tasks	7	(20)	5	(11)
Personal	Demonstrating self-sufficiency in dressing, hygiene, and health	2	(6)	1	(2)
Socialization		26	(74)	39	(89)
Interpersonal	Responding and relating to others including friendships, caring, social appropriateness, and conversation	18	(51)	33	(75)
Play	Engaging in play and fun with others	2	(6)	3	(7)
Coping	Demonstrating behavioral and emotional control around others	9	(26)	15	(34)
Motor		5	(14)	14	(32)
Fine motor	Using hands/fingers in daily life	0	(0)	1	(2)
Gross motor	Using arms and legs in daily life	5	(14)	13	(30)
Themes in caregiver narratives					
Agency		14	(40)	22	(50)
Independence	Behaving without prompting	9	(26)	13	(30)
Confidence	Demonstrating confidence in abilities or ideas	2	(6)	8	(18)
Courage	Acting bravely; different than confidence in that the child acts despite danger, fear, or anxiety	6	(17)	9	(20)
Prosociality		19	(54)	29	(66)
Empathy	Perceiving others' feelings	7	(20)	15	(34)
Generosity	Sharing an object or opportunity that is meaningful to them	3	(9)	6	(14)
Helpfulness	Helping others	10	(29)		(23)
Protecting family	Acting to keep a family member safe	3	(9)	3	(7)
Accomplishment			(34)	31	(70)
Academics	Performing well in one or more subjects	3	(9)	16	(36)
Athletics	Performing well in sport or dance	4	(11)	13	(30)
Achieving	Working to succeed with a given task or activity	6	(17)	18	(41)
Overcoming	Overcoming a previous obstacle, improved on a weakness; different than achieving goals because child faces a barrier to success	7	(20)	6	(14)
Obeying rules	Following rules set by others	5	(14)	4	(9)
Religious faith	Demonstrating belief in God	0	(0)	3	(7)
Settings in caregiver narratives					
Home	At home	18	(51)	24	(55)
School	At school or school functions	9	(26)	22	(50)
Other	At community locations such as church, parks, malls, and events	18	(51)	30	(68)

*Note.* The values in each general category do not equal the total of the values in the subcategories because a given case could include more than one example that fit the category. For example, a parent who emphasized the child's accomplishments may have done so by sharing examples of both academic and athletic success. DLD = developmental language disorder; TD = typical language development.

#### Agency

Agency was a theme that occurred in roughly half of the proud caregiver narratives in the DLD group and the TD group (see Table 2). In both groups, caregivers often remarked on their children's ability to behave independently. For the DLD group, examples of agentive behavior often reflected their strengths in the daily living subdomains of domestic function (e.g., completing chores or preparing a snack) or personal function (e.g., bathing or dressing), as exemplified here:

Girl with DLD 97:

*“Um the musical like this year she had a musical—and what do they call it the winter program I guess—at school. And she was just so excited like she had to get all dolled up and like she just actually participated and like she was all for it and like she was a little shy at first but you know…she was extremely happy. [Examiner: What made you proud with that?] Just that she took initiative like, she dolled herself up, she wore a dress, you know she got herself ready.”*

Notice that this child's agency in the home environment was contrasted with her need to overcome shyness in the public environment (“she just actually participated,” “she was a little shy at first”). Overcoming internal obstacles to achievement was a theme that characterized 20% of the narratives in the DLD group.

The following narrative is rich with revelations about this child's strengths. First, the event itself illustrates his abilities in two developmental subdomains: domestic (doing chores) and play/leisure (planning a play activity). Moreover, in addition to the agency he demonstrated by independently deciding to sacrifice his desired toy, he demonstrated prosociality in his generosity to others, a theme we further illustrate below.

Boy with DLD 139:

*“Uh, he's always done chores around the house and he's always earned his own money and that day he earned his fair amount of money and I took him to the Dollar General to pick out some toys and he decided, he found like a fire truck he wanted, and then he grabbed like three sticky hands and I'm like, ‘Why do you need three of these A? Why, why would you need three of these?’ He's like, ‘So the girls can have one.’ It was when I was baby sitting two other little girls that were the same age as him. Both of their names were B. It was B, B, and A that I watched. And he didn't have enough money in his wallet to get the fire truck and the sticky hands so he actually put the fire truck back just so that he could buy him and the girls all the same sticky hands so they could play together. And it was just really sweet cause he really wanted the fire truck but, he made the decision to get something, get one of each for them so they could all play together.”*

In other narratives, caregivers described their children as courageous; the child acted despite danger, fear, or anxiety. These narratives also provided converging evidence of coping as a strength for many children with DLD.

Girl with DLD 4:

*“…her going through like all her surgeries and stuff like that too, so that was a huge thing. Just being able to at 3-years-old be able to conquer that, and then when her brother was born like she like. When he was born, she had her surgery at like a month later on her eyes again. Like, she had to get more scar tissue built up removed and her just her courage at like 5 or 6 to be able to go through that.”*

Boy with DLD 39:

*“…the day we moved, our house wasn't ready we stayed at my parents', and that night he for the first time sobbed and sobbed but at the end he said, ‘I know I’m sad but I'm still a little bit excited and I'm going to try really hard to make new friends.' And so just had a really good perspective through all of it, probably better than any of us honestly. So and he's done a really great job of plugging in and extending himself and you know working hard because he jumped in mid school year which was challenging, new teacher, new school, new friends, new everything. So he's done a really fantastic job.”*

#### Prosociality

Prosociality was the most frequent theme in the proud caregiver narratives of the DLD group and the second most frequent theme in the narratives of the TD group (see Table 2). Although we saw an example of the generosity subtheme in the narrative about child 139 above, the subthemes of empathy and helpfulness were more common.

In the following example, a child with DLD demonstrates empathy for a classmate, someone he “really wants to know and understand.” The narrative also illustrates this child's strength in the play/leisure domain (desire to initiate play) and his agency in deciding to befriend the girl. He “chooses” to do it, and not because “he should.” This narrative is also among the few from the DLD group that emphasized positive interpersonal relationships with friends.

Boy with DLD 105:

*“Well okay so like in his class he's had this little girl in his classroom every year, well he's only going into second grade. Her name's A and she has Down syndrome and he's amazing with her. And he's not amazing with her in a way that like some kids will do it because they think, you know some like kids get lots of extra attention like A because she's got a lot of people who help her and some kids will kind of fawn on her. But B like this real friend with her. Like he'll choose to play with her, he chooses to want to sit by her. And not for you know I mean he should. You know, A, is a friend in the classroom. But I just think he's really aware of her, like her, you know he'll talk about her like A did this and why does A do that? And you know he really wants to know and understand. And she just thinks he's amazing. I'm really proud of him for that.”*

In the following narrative, the child's prosociality is expressed as helpfulness to her mother. Here, too, she offered this kindness independently, not because someone asked her to; thus, we again see agency presented as a strength. This interview was an exception in that the child herself was present; hence, the mother addressed both the girl and the examiner.

Girl with DLD 13:

*“When you went out there in the rain and you fed the chickens because I didn't want to. And you sucked it up and you went for me. [Examiner: Can you tell me a little bit more about it, like what specifically made you proud. Child: I really don't remember that time.] It was like pouring down, like pouring, like waterfall, and man I have to feed these stupid chickens? I'll do it for you mom! Really? Here. And she did, she came back she was all wet, you were soaking wet. She sucked it up and did it for me.”*

#### Accomplishment

Although the DLD caregivers were similar to the TD caregivers in the frequency with which they emphasized agentive and prosocial behaviors, they emphasized accomplishments less often (see Table 2). Moreover, when they did share accomplishments, they tended to describe internal obstacles the child had to overcome before achieving success. In contrast, for the TD caregiver group, the nature of the accomplishments often referred to a measurable achievement or something that meant the child stood out in a recognizable way to others, such as being the best at reading or sports. The story lines below are remarkably similar: Both children dealt with a difficult coach, and both were good at sports (and indeed both scored above average on the gross motor subdomain of VABS-3). However, notice how the boy with DLD had to overcome his shyness, whereas the girl with TD demonstrated no internal barriers. In fact, she not only played well, she was also confident enough to lead her teammates in play.

Boy with DLD 104:

*“Probably gosh the first time he did a extracurricular activity and he, like I said, was so shy and quiet and so it was microsoccer, and he had a very tall and outspoken coach and all new people he didn't know. And so the very first time he did not want to go out on that field even to warm up or anything. So I think when he actually did get out there then like the second week and played and did great, scored a goal you know like. That was, that was pretty awesome and cheering him on.”*

Girl with TD17:

*“…But she's just really good at soccer. And I was proud of her because this year for soccer she was the only girl on an all-boys team. Um she was for sure the best player on the team and I'm not just saying that because I'm her mom…So I just think I'm proud of her because the coach she had, um, according to coaching standards, was terrible…I just was proud of her for sticking in it. She would actually try to pass and like try to involve her teammates a little bit more. Um, but it just got to the point where we were all like, just score, I'm like you should just shoot and score. But I was just proud that she would like get—like then she'd go to soccer, she wouldn't always want to go, but then she'd go to the game and she'd be like, Mom I scored four goals, and she was just so proud of herself.”*

To summarize, given three strands of evidence, VABS-3 relative strengths and weaknesses profiles, VABS-3 norm-referenced scores, and the proud caregiver narratives, children with DLD tended to demonstrate strengths in the domestic and personal aspects of daily living, the coping and play/leisure aspects of socialization, and gross motor function. The proud caregiver narratives further suggest that broader domains of agency and prosociality are areas of strength for many children with DLD, as predicted. We turn now to a consideration of their weaknesses. We used the VABS-3 data and the case history data as sources. Somewhat counterintuitively, the proud caregiver narratives also provided relevant data in that they sometimes juxtaposed a child's strengths and weaknesses. We share this narrative as an example and a transition to the consideration of weaknesses among children with DLD.

Boy with DLD 72:

*“Oh geez. I mean he makes me proud everyday there's always something that he does that will make me proud. Um it's just I just can't…there's always something, you know. Well, soccer. Uh when he scored a goal at soccer. He was happy, we were happy, you know it was something that he accomplished, he wanted to do, he really likes to do it. Um…things like that.*

*Um school is more of a struggle for him.*

*And another thing that I am very very proud about is that he's learning this year to kind of control his outbursts, um behaviors, you know, he's not on the behavior program this year which is a big, big step for him because last year was awful. But um that's a big one right there. That's it.”*

### Weaknesses

#### Communication

Functional communication was a clear weakness for the children with DLD, in both relative (see Figure 1) and normative (see Table 1) terms. Written communication tended to be the subdomain of greatest weakness; 11% of children with DLD presented with a writing disability, and an additional 29% presented with subclinical weakness. None of the children in the TD group had a disability in any communication domain.

The case history provided converging evidence for the high rate of communication weaknesses and disabilities within the DLD group. Specifically, 49% of children with DLD were reported to show signs of communication avoidance or frustration in everyday life. This compares to 7% of children with TD, χ^2^(2, *N* = 79) = 17.97, *p* = .00002. The children with DLD who presented with avoidance or frustration included nine of the 11 who scored as having a communication disability and eight of the 10 children who presented with subclinical communication weaknesses on VABS-3.

Proud moments involving the communication domain were rare among the caregivers in the DLD group and about 4 times less frequent than in the TD group (see Table 2). However, two DLD caregivers focused on overcoming communicative limitations. In the first, the child's frustration over failed communication is apparent, as is the mother's desire to understand. The mother is proud of his growing ability to successfully reflect on the problem and manage his reactions to it. Thus, we see a weakness (expressive communication) contrasted with an emerging strength (coping).

Boy with DLD 26:

*“I think it was probably after one of the times that he really got upset and couldn't articulate, and he just kind of threw a tantrum. And he came back and apologized, and he just said, ‘Mom I don’t understand why I do that sometimes…I couldn't find the words and I got so frustrated. So I'm sorry I did that, I don't know, I'm gonna try to be better.' He had some reflection and it really helped me get kind of insight into that, what's going on in his mind when he does do that. I was really proud of him.”*

In the second instance, the mother's relief when the child was finally able to communicate something important via words is palpable. She realizes that most caregivers can take such developments for granted. It should “just be” and “on most kids it is,” but she and her son have had a different experience.

Boy with DLD 94:

*“I was very proud of him the day he was able to tell me his ear hurt. He was able to tell me his ear was owie, and like his ear hurts, and I'm like, he had an ear infection. And I was able to know, he was able to tell me that, like, he was able to tell me a feeling of his that isn't visual. Like, he's got an owie. His ability to talk is the thing that I'm the most proud of. And that's something that I shouldn't be proud of, because something should be just be, a concept that's fact. On most kids it is. On mine, I'm proud that my kid can talk to me.”*

The importance caregivers place on their child's healthy communication development was also illustrated by this narrative about a girl from the TD group. This child has typical language skills, as evinced by a standard score of 103 on the TNL-2, and good communicative function as evinced by a standard score of 100 on the VABS-3 Communication domain. However, she was described as shy, and her reticence to speak (or perhaps her difficulty with articulation) upon entry to kindergarten had been a great source of concern to her caregivers.

Girl with TD 42:

*“…Well, I'll say this. Kindergarten. My baby wasn't like on level far as speech-wise, because she wasn't like around—she was the baby, so she wasn't around like a lot a little kids. So we got into kindergarten, and she was like a little standoffish. She was nothing like she is now. She wouldn't like really talk because her speech wasn't there. And then she was like a little standoffish. Well about the end of the year you know you do that little graduation or whatever, she was singing them songs, and everybody was her friend, and she had just did a total transformation. And I was say, ‘Oh look at my baby!’ And I still be watching the video sometimes because she was just like in her—just like a little glow on her. You know. And I was just saying okay she came out of her shell, but, even her speech and everything was like better, and, she was wanting to learn. Whereas when she first started it was like, I'm just here, y'know, I'm gonna' go with the flow, but I want to be invisible. And now she's like, she's, she makes sure she's always in the front: ‘See me, know me.’”*

With the exception of child 42, the focus on communication-related moments among the TD caregivers was related to their children's academic achievement or prosocial behavior toward their siblings with regards to reading and writing, as illustrated in the two excerpts below. These sorts of examples never occurred within the narratives of caregivers in the DLD group.

Girl with TD 43:

*“…You know so one thing I think a few months ago she won a writing award and so I was incredibly proud of her. She had to get up on stage and accept her award and I just knew that meant a lot to her because she wants to write and it was a good boost for her confidence and things too…the thing I don't think is normal, like how much she enjoys that I don't know (laughter) like most kids. She asked for notebooks for Christmas so she could write stories (continued laughter). It just it cracks me up.”*

Boy with TD 46:

*“…Or like with reading, like, you know all of a sudden like at night he can read to A a book, pretty cool. Like at night he'll read, he'll ask now to read her bedtime story so that's sweet. That's proud, like you know you're glad that he wants to like read to her.”*

#### Community Aspects of Daily Living

Community aspects of daily living were challenging to many with DLD, as evident by the number of children who had a relative weakness in that area, a lower group mean performance compared to their TD peers, and an 11% rate of disability (see Figure 1 and Table 1). In fact, outside of the communication domain, the largest gap between the DLD and TD groups on VABS-3 was in the subdomain of community function. In both the DLD and TD groups, proud moments often occurred in the community—parks, auditoriums, and stores were frequent settings. However, they did not involve community function as tapped on VABS-3.

#### Interpersonal Aspects of Socialization

As a group, children with DLD scored significantly lower than their TD peers on the interpersonal subdomain of VABS-3; however, none of the children with DLD scored so low as to be considered disabled. It is obvious that their caregivers placed high value on their children's interpersonal relationships given that half of their narratives included examples of positive ways that their children relate to others. However, when examined closely, those frequent examples are not contrary to the finding of low interpersonal function on VABS-3. The DLD caregivers overwhelmingly emphasized caring relationships with family members rather than friends, for example:

Boy with DLD 61:

*“…he's always really good with like his younger siblings which he's always looking out for them or and encouraging them, helping them ride a bike, yeah um protective of (th)em.”*

Boy with DLD 124:

*“…Um I would probably say like when he helps his brother out with certain things or he encourages or you know tells his brother, ‘good job,’ or you know helps him get something. I mean that's, I always tell him you know you guys have you know you're brothers and you always have to be there for one another. So, I, I mean that always makes me proud when he's super nice to him and you know encourages him and is you know always, always there for him basically so, that makes me happy.”*

In fact, only six DLD narratives even included the word “friend.” Questions on the VABS-3 interpersonal subdomain, in contrast, probe relationships with friends more than family. They also tend to be heavily language focused (e.g., does the child engage in small talk or recognize that a communication partner needs additional background information).

#### Academics

Thirty-seven percent of the children with DLD had an academic disability, and an additional 26% had weaknesses, for a total of 63% (see Table 3). The most common profile involved below-average academic performance together with difficulty completing school assignments. None of the children in the TD group demonstrated academic disabilities or weaknesses.

**Table 3.** Academic function of children with DLD and TD.

Function		DLD *n* = 35	TD *n* = 44
Disabled		13 (37%)	0
	Below average academic performance and difficulty with school assignments	9	
	VABS-3 written language score < 10	1	
	Both of the above	3	
Weak		9 (26%)	0
	Below average academic performance	4	
	Difficulty with school assignments	2	
	VABS-3 written language score = 10 or 11	3	
Healthy	None of the above	13 (37%)	44 (100%)

*Note.* DLD = developmental language disorder; TD = typical language development.

The caregivers' impressions of their children's learning abilities provided converging evidence of the academic difficulties among the DLD group. The impressions of the DLD caregivers were distributed fairly equally, with 23% being positive (“strong memory, eager to learn”; “I would say he has no poor subjects”), 29% being negative (“will need help”; “takes her longer to process information”), 23% being mixed (“she is capable but just needs more time”; “I believe she is still learning and will get to where she needs to be”), and 26% being no response. In contrast, the majority of caregivers of children with TD (77%) shared positive impressions, and some were extremely positive (“he learns very quickly, I believe he is gifted”; “quick study, very easy for him”). The distribution of responses differed by group, χ^2^(2, *N* = 79) = 25.64, *p* = .000011.

The struggles children with DLD faced with academics were evident in the proud caregiver moments, not by their presence but by their absence. The school was the setting for about half as many proud caregiver moments in the DLD than TD group, χ^2^(2, *N* = 79) = 4.8, *p* = .03. Only three (9%) of the DLD narratives involved academic achievements (see Table 2).

To summarize, given three strands of evidence, VABS-3 relative strengths and weaknesses profiles, VABS-3 norm-referenced scores, and the proud caregiver narratives, children with DLD tended to demonstrate weaknesses in all domains of communication, the community subdomain of daily living, the interpersonal subdomain of socialization, and academics. In some but not all children with DLD, weaknesses were extreme enough to constitute disabilities. We turn now to a more complete consideration of these children.

### Relationship Between Disability, Impairment, and Risk

**Figure 2.**
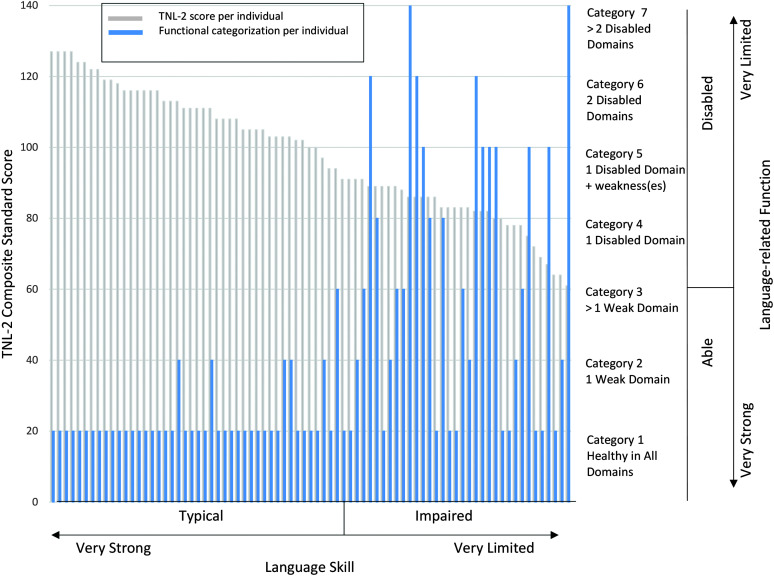
The relationship between language skill as measured by the Test of Narrative Language–Second Edition (TNL-2) and language-related function.

Figure 2 aligns language skill (*x*-axis) with language-related function (right-side *y*-axis). Specifically, each participant has a gray bar representing their score on the TNL-2 and a blue bar summarizing their overall performance over the primary domains of communication, daily living, socialization, and academics.

First, consider the children with typical language skills. Most had healthy function; five demonstrated a subclinical weakness in one domain, and one (who was one of two with the lowest language skills in the TD group) demonstrated a subclinical weakness in two domains.

Second, consider the children with impaired language skills. In the ICF biopsychosocial model, impairment alone is not sufficient for the demonstration of disability. Our data support that model: 11 out of 35 (31%) presented with healthy function in all domains, 10 (29%) presented with one or more subclinical weaknesses (but no disabilities), and 14 (40%) presented with one or more disabilities.

When we combine the TD and DLD groups to consider language skill as a continuum, we find a significant relationship between skill and function, as predicted. Stronger skills as measured by the TNL-2 are associated with better function (i.e., lower category numbers). A Spearman's rho test for ordinal data confirms this relationship, *r*_s_ = −.58066, *p* (two-tailed) < .05. However, within the DLD group, there was no significant relationship between skill and functional classification, *r*_s_ = −.04383, *p* (two-tailed) = .80258. In eight instances, children with identical levels of language impairment presented with different levels of function (see Figure 2 and Table 4).

**Table 4.** Language skill, language-related function, risk factors, and receipt of language services among participants with DLD.

ID	Language skill	Language-related function				Risks			Language services
	TNL-2 SS	Comm.	Acad.	Daily living	Social	NVIQ	SES	Other health condition	
81	91						L		
86	91						L		
136	91		W			L		Heart	SLP
26	91	W	W						
97	89	D	D			L	L		
13	89		D			L	L		
71	89						L		SLP
59	89		W					Feed/Swallow	SLP
72	89	W	W		W			ADHD, Sleep, Behavior	
93	88	W			W	L		ADHD, Feed/Swallow, Motor	
121	86	D	D	D	W	L	L	Behavior	SLP
68	86	D	D			NA			OP
41	86	D	W					ADHD	SLP
55	86		D						OP
98	86					L			
82	83		D					Motor, Depression, Anxiety	SLP
39	83								SLP
75	83								SLP
90	83	W	W			L			SLP
33	83		W						
4	82	D	D					Vision	OP
104	82	W	D		W				OP
69	82	W	D			L		Feed/Swallow	OP
61	80	W	D						SLP
21	80								
124	78								
63	78			W			L		
127	78	W	W	W	W			Skeletal	SLP
94	75	W	D	W				Motor	SLP
52	72								SLP
116	69					L			
137	67	W	D			L		Motor, Sensory Processing, Balance	SLP
139	64							ADHD	
141	64		W				L	ADHD, Heart	
103	61	D	D	D		L		ADHD	SLP

*Note.* DLD = developmental language disorder; TNL-2 SS = standard score earned on Test of Narrative Language–Second Edition; Comm. = Communication; Acad. = Academic; NVIQ = nonverbal intelligence quotient; SES = socioeconomic status; W = subclinical weakness; D = disability, L = low (for NVIQ low = standard score of 70–84; for SES, low = < 13 years of education for most educated caregiver); NA = not available, ADHD = attention deficit-hyperactivity disorder, SLP = speech-language pathologist providing service, OP = other professional providing service. Blanks = healthy function, NVIQ scores of 85 or higher, parents with higher than 12th grade education, no chronic health conditions other than DLD, and no receipt of services.

Table 4 presents the details of the functional profiles of each child in the DLD group along with the risk factors we tracked. Twenty-seven of the 35 children with DLD (77%) presented with at least one risk factor. What appears to differentiate functional levels is the accumulation of risks. Among the 24 children with DLD who presented with weak or disabled function, 11 (46%) experienced two to four risk factors (counting each health condition separately). None of the 11 children with DLD who had healthy function had more than one risk factor.

### Disability and Receipt of Services

Overall, 20 of the 35 children with DLD (57%) received services from an SLP or other language-related professional, such as a reading specialist (see Table 4). Among the 24 children with functional disabilities or weaknesses, 16 (67%) were currently receiving services. Among the 11 who presented with healthy function, four (36%) were currently receiving services.

The children who faced challenges to healthy function but were without services are a concern. If we set aside subclinical weakness and consider only the 14 children who presented with one or more disabilities, we find two children, child 97 and child 13, without support from an SLP or other language-related professional. These girls had mild impairments and were from families with low caregiver education. We will explore these factors further in the Discussion section.

## Discussion

We used a mixed-methods approach to derive a profile of strengths, weaknesses, and disabilities among first graders with DLD. In the following sections, we integrate the resulting profile into the literature, discuss the extent to which the patterns of disability fit the ICF biopsychosocial model, and explore potential barriers to service delivery for children with DLD who present with disabilities.

### What Strengths Characterize Children With DLD?

#### Socialization: Prosocial Behavior, Play, and Coping

As predicted, these first graders presented with particular strengths in socialization. As a group, they scored at age-appropriate levels on the VABS-3 play and leisure, a subdomain that tapped behaviors such as sharing toys with others, demonstrating good sportsmanship, and planning play activities. Moreover, their caregivers shared many examples of their empathy and helpfulness to family members, peers, and adults in the community. Overall, our findings accord with the previous conclusion that children with DLD tend to demonstrate prosociality (Conti-Ramsden & Durkin, 2015). This conclusion is also consistent with that of Shevell et al. (2005). Within their cohort of children with DLD, the rate of disability in socialization was the lowest of any domain.

In this study, children with DLD also scored at age-appropriate levels in the coping subdomain of socialization. These items had to do with regulating emotions and behaviors toward others. Although emotional regulation has been described as problematic for children with DLD (Croteau et al., 2015; Fujiki et al., 2002), our findings might not be completely contradictory. Specifically, the caregiver narratives often emphasized the recent emergence of coping skills. For example, a girl could not separate from her parent without crying during kindergarten, but by first grade, she did so without incident. Another child had problems with emotion regulation that were severe enough to require a behavior intervention program, but he had “graduated” and was functioning well. Growth in coping skills may contribute to resilience in these children (Rutter, 2006).

#### Agency: Independence in Domestic and Personal Aspects of Daily Life

Also, as predicted, agency emerged as a strength among the children with DLD. Their caregivers were proud when their children behaved well without prompting. Good behavior tended to map onto activities of daily life in the home, either domestic, such as doing chores or locking the front door for safety, or personal, such as grooming and preparing simple snacks. As a group, they scored at age-appropriate levels in domestic and personal function, and personal function in particular was a relative strength for over 40% of the DLD cohort. Teachers of children with DLD in the study of Croteau et al. (2015) rated personal function as a strength in the school environment as well, as evidenced by using locker storage appropriately and changing clothes when soiled.

#### Gross Motor Function

Gross motor function was an additional strength among many in the DLD group. Given the high comorbidity of DLD and developmental coordination disorder, we did not expect such robust performance at the group level. It could be that children with DLD have more fine motor than gross motor problems or more specific problems like oral motor coordination (Malas et al., 2017) and bimanual timing (Vuolo et al., 2017) deficits. It also could be that gross motor impairments did not present functional limitations in this study because the communities we sampled valued sports training and activities for children highly. Although athletic accomplishments were not emphasized by the DLD caregivers as often as the TD caregivers, many of the “overcoming” themes expressed by the former had to do with gross motor mastery—children finally hitting a ball, completing a tumbling routine, or swimming the length of the pool—after numerous practice opportunities.

### What Weaknesses Characterize Children With DLD?

Whether measured relative to their own strengths or relative to age expectations, communication, the community functions of daily living, the interpersonal aspects of socialization, and academics were the primary areas of weakness and disability for the children with DLD. Academic function was most often disabled (but note that it was not measured with the same instrument as the other domains), followed by communication, community functions, and interpersonal relationships. Not coincidentally, high language demands infuse each of these domains.

#### Academics

Academic difficulties were highly prevalent: Given parent reports, 37% presented with academic disability. Caregivers of children with DLD were less likely than caregivers of children with TD to talk about their children's emergent reading and writing and less likely to situate their proud moments in the school setting or share examples of their child's academic achievements. These findings accord with an extensive literature documenting academic challenges among children with DLD. Norbury et al. (2016) found that 88% of children with DLD identified via community sampling did not meet academic milestones in first grade via teacher ratings, and Dockrell et al. (2011) found that the academic difficulties of children with DLD persist throughout the school years.

#### Communication

Understanding directions, telling about a recent experience, or writing simple sentences were difficult feats for these children. Written communication was weaker than spoken communication, a finding also reported by Croteau et al. (2015). The only surprise here may be the relatively low rate of functional communication problems. Communication was a disability for 17% of the children with DLD in our sample but 48% in that of Shevell et al. (2005). Recall, however, that theirs was a clinical sample, but ours was not. It may well be that signs of communication disability lead caregivers to seek a clinical evaluation.

#### Community Aspects of Daily Life

Daily life activities in the community were disabled in 11% of the children with DLD and a point of weakness for an additional 14%. Child-appropriate community items on VABS-3 included obeying walk signs, using the phone, and making purchases at a store. In the study of Croteau et al. (2015), parents of children with DLD also reported that the use of money and respect for safety guidelines were problematic for their children. Community functions are not only language laden but also experience dependent. The extent to which caregivers allow children with language challenges to have independent experiences in the community, or restrict those experiences to ensure their safety and success, is an open question. However, the parents of children with DLD who were interviewed by de López et al. (2021) reported extensive scaffolding of their children's interactions with community members (e.g., helping them to order at the bakery).

#### Interpersonal Aspects of Socialization

Despite their tendency toward prosociality, the children with DLD did not score well on the interpersonal relationship subdomain of VABS-3; however, none scored so low as to indicate disability. In fact, their weakness in this domain was fairly specific. The caregivers of children with DLD tended to describe positive relationships with family members, especially child and parent or child and sibling. Although they mentioned kind actions toward peers, like helping a child who had fallen, they rarely talked about friends or friendships. Loneliness, peer rejection, and problematic friendships among children with DLD have long been documented. In a recent examination of social networks in kindergarten classrooms, the odds of having a reciprocal friendship were 50% lower for children at risk for DLD than for other children (Chow et al., 2021). Relationships with peers among teens with DLD may be even more problematic as adolescence brings new challenges to social function (St Clair et al., 2011).

Problematic peer relationships could directly reflect language limitations that cause interpersonal interaction to falter. For example, parents report that frequent communication failures make it difficult for their children with DLD to establish and maintain friendships (de López et al., 2021). Associated deficits in emotion regulation could also contribute to problematic peer interactions (e.g., Fujiki et al., 2002). A child's communicative limitations may be more accepted and better adapted to within families than among peers or unfamiliar partners (McLeod et al., 2013; Van Doornik et al., 2018).

### Factors That Contribute to Healthy and Disabled Function

According to the ICF, it is theoretically possible for someone without an impairment to demonstrate a disability (e.g., under extremely harsh conditions), but in this study, no child with typical language skills presented with a disability in the primary language-related domains of communication, daily living, socialization, or academics. In contrast, function among the children with impaired language skills was mixed: Some were disabled, others had one or more subclinical weaknesses, and others still had healthy function. Although it is possible that some of the healthy children were false negatives, we think it doubtful given that VABS-3 is well validated and has been found to be a better predictor of the (academic) function of children with DLD than traditional decontextualized tests of language skill (Hall & Segarra, 2007). Instead, in accordance with the ICF model and the findings of Shevell et al. (2005), we conclude that not all children with language impairment present with disabled language-related function.

Moreover, the severity of language impairment as measured by decontextualized tests of language skill was an imperfect predictor of disability. At the case level, there were eight instances of two or more children with equivalent levels of impairment but differing levels of function. What instead differentiated functional levels was the accumulation of risks. It was not whether the child had a low NVIQ, was from a family with low caregiver education, or had various other health conditions; the majority of the children with language impairment presented with at least one of these risk factors. Instead, where function was concerned, it seemed to be the accumulation of factors that mattered. None of the 11 children with DLD who had healthy function had more than one of the risk factors we tracked. Cumulative risk predicts the presence of DLD itself (Whitehouse et al., 2014) as well as broad current and future developmental outcomes in academic and health domains (Atkinson et al., 2015).

### The Relationship Between Disability, Impairment, and Receipt of Services

#### Not All Children With DLD Require Special Services

According to U.S. federal law, receipt of services among those with impairments should hinge on the presence and extent of disabled function, and we found that it did, to a degree. The majority of the children with DLD and healthy function were without services from an SLP or any other professional. The lack of services in these cases may be sensible. When children function well, we do not want to pull them away from classroom experiences, family life, play, rest, or other valuable activities to participate in structured interventions. Moreover, we want to reserve the scarce resources of time and money for children who need external supports. However, we qualify this statement by emphasizing the developmental nature of neurodevelopmental disorders. Children who are impaired but not disabled at any given time should be monitored in case disabilities arise as contexts change and demands on function rise.

#### Mild Levels of Impairment May Be a Barrier to Receipt of Services

Although best practice is to avoid arbitrary cut-points on standardized tests when determining the presence of an impairment (Plante & Vance, 1994), some states in the United States continue to consider −1.5, −1.75, or − 2.0 *SD*s below the mean as criterial regardless of the tests being used (e.g., Alabama Administrative Code, 2022; Louisiana Administrative Code, 2017; Missouri Department of Elementary and Secondary Education, 2020; Tennessee Department of Education, 2017). In the current sample, only seven of the 35 children with DLD scored lower than −1.5 *SD* on the TNL-2 and thus would have qualified for services given these arbitrary cutoffs. To paraphrase Fletcher and Miciak (2019, p. 22), there is no natural demarcation between scores indicative of DLD and typical development. Districts should not use formulae or strict thresholds as a gatekeeper for identifying and thus serving children in need.

The two disabled children who lacked services were girls. Girls with DLD are less likely to be identified than boys (Calder et al., 2022; Morgan et al., 2017; but see Norbury et al., 2016). Although we have been aware of this inequity for decades (Zhang & Tomblin, 2000), the reasons are poorly understood. Language impairment itself could present differently in girls than boys at the neural (Ullman, 2016) and behavioral (McGregor et al., 2020) levels. Also, girls may present with different strengths or strategies than boys (Hart et al., 2004) that mask their language difficulties to a greater extent. Finally, communication partners (e.g., teachers, peers) might respond differently to girls than boys who have DLD (Chow et al., 2021), thereby reducing the extent of their language-related disability in certain contexts.

We also note that the two disabled children who lacked services had caregivers with less than 13 years of education. Although school-based speech-language services are free to those who qualify under IDEA, caregivers with low levels of education or income face obstacles to receipt of those services (Bishop & McDonald, 2009; Wittke & Spaulding, 2018). The hidden costs for families include the time and money required to work out the logistics of assessments and the planning of the Individualized Education Program. Moreover, families with low educational attainment may also lack the cultural capital that higher education enables—the professional jargon, scripts, and credentials that enable affluent families to navigate the bureaucracy inherent in realizing one's rights under IDEA (Skrtic et al., 2021; Terry et al., 2022). And when will that lack of cultural capital matter most? When the child has a mild impairment and thus does not meet arbitrary cutpoints for qualification.

### Clinical Implications

#### Prevention and Surveillance

At any given point in development, a given child with DLD may function well at school, with family, and in the community. At that time, the child's internal strengths and external environment are adequate compensation for their impaired language skills. Under IDEA, this child does not need at-school services from an SLP or other language professional. That said, disability is dynamic. Thus, it is critical to participate in prevention programs such as Response to Intervention that might stave off disabled function and to maintain surveillance of children dismissed from speech-language services in case disabled functions re-emerge. Those who have been dismissed may need to rejoin a caseload later, not because they have lost ground in any absolute sense but because they are facing new challenges.

#### Qualification

In compliance with federal law, U.S. school districts uniformly recognize that only children with disabilities—problematic function in the educational environment—should receive speech-language services. School SLPs routinely use functional measures such as language samples, classroom observations, and teacher interviews to identify these problems. However, the presence of language impairment is also prerequisite to service receipt and non–evidence-based practices for the identification of language impairment codified in some state guidelines may result in some disabled children falling through the cracks. Specifically, our results demonstrate that the severity of the language impairment is not always a reliable predictor of the extent of disability. Some children with mild impairments function poorly. Districts that require arbitrary cutpoints on standardized tests of language skill to identify impairment will not qualify these children for services.

To ensure that all children with language-related disabilities are served, we should abandon arbitrary cut-points on standardized tests and instead use test-specific cutpoints that maximize sensitivity to language impairment (at any level of severity). Arizona is among a handful of states that illustrate this best practice (Arizona Department of Education, 2016). As we await improved policies in other states/districts, savvy SLPs may avail themselves of alternatives to standardized tests, a practice permitted in many districts.

#### Treatment

Knowing that disability resides as much outside of the child as within, we may entertain new directions for treatment. The risks that we considered here, low NVIQ, low caregiver education, and other health conditions, are not highly malleable. However, some risk factors are. For example, when interviewed about what an ideal language intervention would look like, 10- to 13-year-old children with DLD focused on aspects of the school environment that posed barriers to their participation and achievement (Gallagher et al., 2019). They wished for a setting in which they felt safe to take risks with learning and talking and in which they had chances to practice talking that extended beyond answering the teachers' questions. In addition, they desired support that would help them understand the implicit rules of school and successful social interactions. These children provide insights into what an intervention should include when the therapist or teacher recognizes that the environment around the child plays a significant role in determining disability.

### Limitations and Future Directions

We mined the case history reports given by the caregivers to ascertain the children's academic performance and the caretakers' educational attainments. These are blunt measures that could be improved in future studies. For example, the Student Language Scale (Nelson et al., 2018) is a survey that has high sensitivity and specificity in the screening of language-related academic function, and Adler et al. (2000) offer a measure of SES that does not depend upon caregiver education as a proxy or require families to reveal their household income levels but rather on the participant's own perception of their SES relative to others.

We interviewed caregivers because of their essential role in supporting their children's development and everyday life functions. However, this particular sample of caregivers was limited to English speakers who live in the United States, primarily in two midwestern states. Other communities may foster different lived experiences and thus different perceptions of children's strengths and weaknesses. A broader sampling would improve the external validity of this line of work.

Moreover, the understanding that we gleaned from listening to caregivers would be further enhanced by other voices. Children with DLD will have authentic insights into their abilities and disabilities that may differ from those of the adults in their lives (Hughes et al., 2009). Teacher and caregiver perceptions may also be complementary (Croteau et al., 2015). Teachers have direct and extensive opportunities to observe the child's functions in the academic context, and these observations can benefit from a built-in basis for comparison, the child's classmates. Given the widespread reports of academic dysfunction among children with DLD, teachers' perspectives will be vital to our understanding of the disabilities that can accompany DLD. In fact, the prevalence of academic disability in this study, as estimated by parents, was substantially lower than the prevalence estimated by teachers in the study of Norbury et al. (2016), suggesting that caregivers may have underestimated the extent of academic disability among children with DLD.

A related limitation of this study is that because we relied upon caregiver reports, we were not privy to the details of the treatments that identified children were receiving. It was clear that professionals other than an SLP were treating some children, but without access to school records, we lacked the details to document the nature and intensity of the services. When presented with case studies that illustrated potential manifestations of DLD, the vast majority of classroom teachers—who are in an excellent position to refer children to be evaluated for service qualification—indicated that they would most likely provide in-class intervention in these cases (Girolamo et al., 2022). The ideal combination of classroom teachers, reading specialists, SLPs, and paraprofessionals required to address the range of functional weaknesses and disabilities accompanying DLD is an essential question for future study.

To some extent, we were encouraged to see that 67% of the children with DLD who had weak or disabled functions were receiving language-related services of some kind, as this compares favorably to other reports that hover around 33% (Norbury et al., 2016; Shevell et al., 2005; Tomblin et al., 1997). However, note that ours was neither a clinical sample nor a proper community sample. Although we screened in the community and advertised broadly rather than pulling from clinic caseloads, the caregivers who agreed to enroll may have been motivated to do so because their children were receiving treatment. Thus, an additional limitation of this study is that we cannot attest to the validity of the 67% estimate. Nevertheless, the critical point is that even though our sample may have been biased toward children with services and even though we took into account the functional level of the children rather than impairment only when identifying service needs, we still found gaps in service delivery.

## Conclusions

In summary, as a group, the children with DLD presented with weaknesses in communication, community function, interpersonal relationships, and academics, domains that are highly dependent upon language ability. On the other hand, they presented with strengths in domestic and personal aspects of daily living, play and coping aspects of socialization, and gross motor function. In addition, caregivers of children with DLD tended to express pride in their children's helpfulness to others, their ability to behave well without prompting, and their ability to overcome obstacles, and at a broader level, these positive behaviors speak to the prosocial, agentive, and resilient qualities of children with DLD.

This group-level profile obscured individual differences. Some children with DLD had many strengths and few, if any, signs of disability or even any subclinical weaknesses; others demonstrated clear evidence of disability. Cumulative risks rather than the severity of the language impairment predicted disability. Not all children with DLD will have language-related disabilities at any given time. It is imperative that we serve those who do.

## Author Contributions

**Karla K. McGregor:** Conceptualization (Lead), Data curation (Supporting), Formal analysis (Equal), Funding acquisition (Lead), Methodology (Lead), Project administration (Lead), Supervision (Lead), Validation (Lead), Visualization (Lead), Writing – original draft (Lead), Writing – review and editing (Lead). **Nancy Ohlmann:** Data curation (Equal), Formal analysis (Equal). **Nichole Eden:** Data curation (Equal), Investigation (Equal), Writing – review and editing (Supporting). **Tim Arbisi-Kelm:** Investigation (Equal). **Alys Young:** Methodology (Supporting), Writing – review and editing (Lead).

## Data Availability Statement

The raw de-identified VABS-3 and case history data are available in the Open Science Framework (https://osf.io/gw3rz/?view_only=4da9260691d549138d309fc7367e5ada), as are the de-identified transcripts of the proud caregiver narratives.

## Footnote

1 DLD goes by many names, including receptive–expressive language disorder, specific language impairment, speech-language impairment, and developmental aphasia. We use the term *DLD* consistently here, but note that the studies we cite may have used a different term and applied different identification criteria.
